# Supplementary material for: Estimating the Burden of Illness of Relapsed Follicular Lymphoma and Marginal Zone Lymphoma in Ontario, Canada
Source: Curr Oncol. 2023 Apr 30;30(5):4663–76. doi: 10.3390/curroncol30050352 (PMC10216939; doi:10.3390/curroncol30050352)
Supplement: Supplementary file 1 [file curroncol-30-00352-s001.zip › curroncol-2313198-supplementary.pdf]

SUPPLEMENTARY MATERIAL: Table S1

Exhibit 1. ICD-O-3 morphology codes for follicular lymphoma and marginal zone lymphoma.

Cancer Type	ICD-O-3 Morphology Codes
**FL**	96903 = Follicular lymphoma, NOS<br>96913 = Follicular lymphoma, grade 2<br>96953 = Follicular lymphoma, grade 1<br>96983 = Follicular lymphoma, grade 3
**MZL**	96893 = Splenic marginal zone B-cell lymphoma<br>96993 = Marginal zone B-cell lymphoma, NOS

Abbreviations: FL: follicular lymphoma; ICD: international classification of diseases; MZL: marginal zone lymphoma

Exhibit 2. Patient selection into follicular lymphoma and marginal zone lymphoma relapse cohorts, Ontario, Canada, 2005–2018.

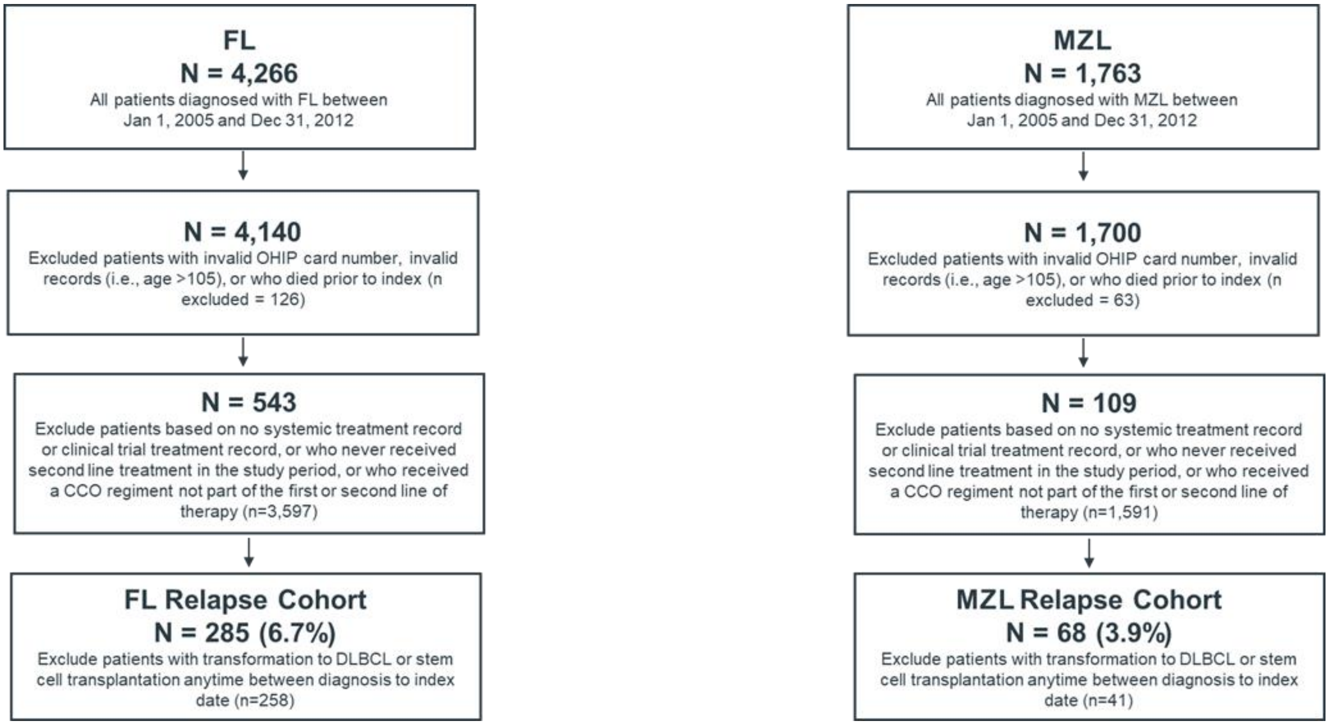

Abbreviations CCO: cancer care Ontario; DLBCL: diffuse large B-cell lymphoma; FL: follicular lymphoma; MZL: marginal zone lymphoma; OHIP: Ontario Health Insurance Plan

Exhibit 3. Mean interval of time from diagnosis to therapy and progression for the A) follicular zone lymphoma relapse and B) marginal zone lymphoma relapse cohorts, Ontario, Canada, 2005–2018.

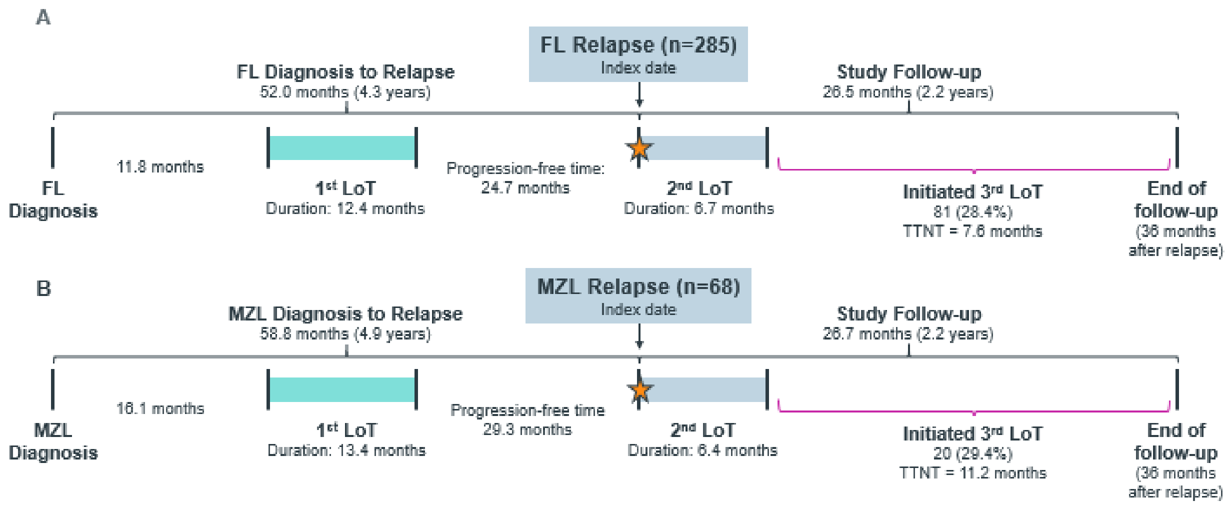

Abbreviations FL: follicular lymphoma; LoT: line of treatment; MZL: marginal zone lymphoma; TTNT: time to next treatment

Exhibit 4. Healthcare resource utilization in the three years following follicular lymphoma relapse by therapy received, Ontario, Canada, 2005–2018.

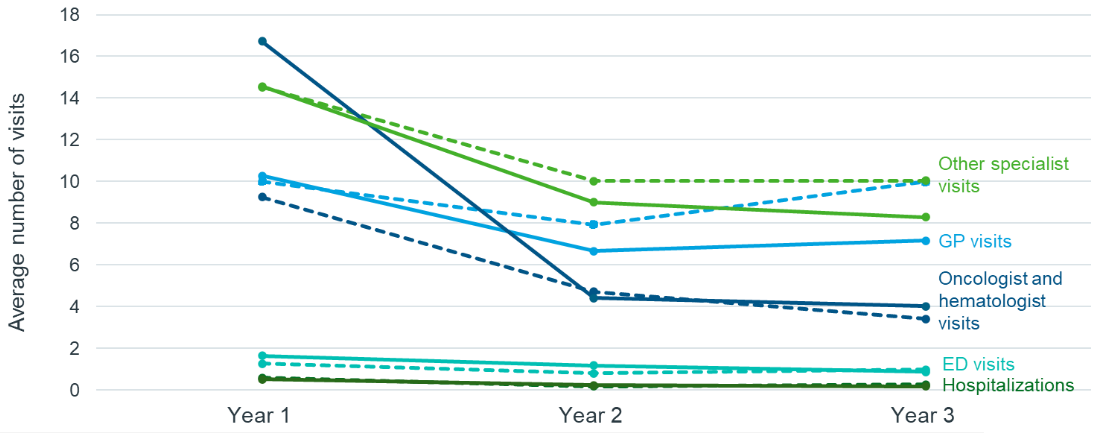

		Year 1	Year 2	Year 3
**R-CHOP/R-CVP/BR used in 1st LoT only**	- -	N = 158	N = *119-123	N = 106
**R-CHOP/R-CVP/BR used in 1st and 2nd LoT**	—	N = 103	N = 83	N = 62

Abbreviations: BR: bendamustine plus rituximab; CHOP: cyclophosphamide, doxorubicin, vincristine, and oral prednisone; CVP: cyclophosphamide, vincristine, and oral prednisone; ED: emergency department; FL: follicular lymphoma; GP: general practitioner; LoT: line of treatment; MZL: marginal zone lymphoma; R-mono: rituximab monotherapy

Exhibit 5. Healthcare resource utilization in the three years following follicular lymphoma relapse for patients who did and did not initiate third-line therapy, Ontario, Canada, 2005–2018.

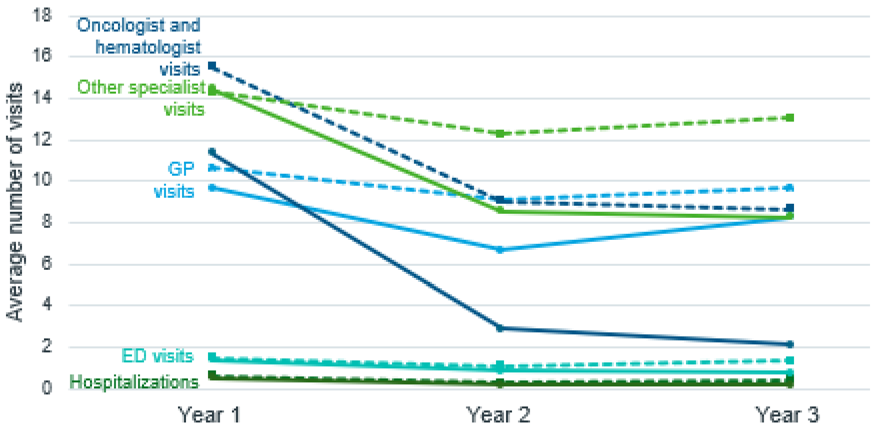

Initiated 3rd LoT	- - -	N = 158	N = *119-123	N = 106
Did not initiate 3rd LoT	——	N = 103	N = 83	N = 62

Abbreviations: ED: emergency department; FL: follicular lymphoma; GP: general practitioner; LoT: line of treatment; MZL: marginal zone lymphoma

Exhibit 6. Proportion of patients with follicular lymphoma relapse and marginal zone lymphoma who made one or more visits for a healthcare service by year post relapse, Ontario, Canada, 2005–2018.

	FL Relapse			MZL Relapse
	**Year 1**	**Year 2**	**Year 3**	**Year 1**
**General practitioner/family physician**	93.0	92.0	90.7	95.6*
**Oncologist and hematologist**	84.2	80.8	76.5	89.7
**Other specialist**	98.9	96.4	90.7	95.6*
**Hospitalization**	35.8	12.5	15.3	36.8
**Emergency department**	56.5	41.1	40.4	58.8

*This proportion estimated based on the median value from a reported range. The actual cell values were repressed to small numbers to reduce the risk of patient re-identification following ICES reporting standards.

Abbreviations FL: follicular lymphoma; MZL: marginal zone lymphoma

Exhibit 7. Average direct healthcare costs (± standard deviation) for patients with follicular lymphoma, who did and did not initiate a third line of therapy, by year post relapse, Ontario, Canada, 2005–2018.

	Initiated a third line of therapy			Did not initiate a third line of therapy		
	**Year 1**	**Year 2**	**Year 3**	**Year 1**	**Year 2**	**Year 3**
	N=81	N=59	N=43	N=204	N=165	N=140
**Any total costs, n (%)**	81 (100%)	59 (100%)	43 (100%)	204 (100%)	*160–164	140 (100%)
	Mean ± SD	*Mean ± SD*	*Mean ± SD*	*Mean ± SD*	*Mean ± SD*	*Mean ± SD*
**Total costs**	$50,281.75 ± $28,956.13	$27,874.53 ± $23,025.79	$28,871.07 ± $31,377.38	$53,343.90 ± $35,239.20	$13,164.87 ± $15,966.06	8,958.25 ± 16,088.99
**GP costs**	$408.68 ± $691.37	$394.71 ± $583.69	$410.21 ± $680.29	$445.61 ± $1,053.08	$241.68 ± $346.70	326.20 ± 637.00
**Oncologist and hematologist costs**	$488.30 ± $880.88	$181.36 ± $372.88	$242.14 ± $868.91	$270.11 ± $530.25	$84.46 ± $175.07	43.36 ± 91.43
**Other specialist costs**	$2,227.23 ± $1,455.10	$2,030.49 ± $1,689.90	$2,152.09 ± $2,064.68	$2,193.33 ± $2,786.65	$1,299.64 ± $1,712.50	1,159.42 ± 1,603.92
**Hospitalization costs**	$5,842.22 ± $8,289.81	$4,058.17 ± $8,486.48	$5,580.79 ± $14,233.32	$6,789.44 ± $19,407.75	$2,269.09 ± $8,260.48	1,673.77 ± 5,708.94
**Same day surgeries costs**	$157.10 ± $577.85	$408.42 ± $1,151.66	$480.30 ± $1,469.36	$274.35 ± $934.59	$254.02 ± $609.90	277.82 ± 846.72
**ED costs**	$553.99 ± $756.22	$497.58 ± $764.27	$519.44 ± $876.22	$557.96 ± $821.08	$301.59 ± $652.38	275.60 ± 498.90
**Cancer clinic costs**	$17,189.42 ± $9,942.96	$7,888.75 ± $7,743.71	$7,305.30 ± $9,810.66	$13,810.92 ± $9,896.94	$1,904.68 ± $2,638.98	938.43 ± 3,999.93
**ODB costs**	$2,996.86 ± $5,102.10	$2,093.93 ± $4,764.96	$1,833.42 ± $3,639.71	$1,847.37 ± $3,001.24	$1,075.04 ± $2,211.27	947.09 ± 1,969.49
**NDFP chemotherapy drug costs**	$13,963.25 ± $17,407.78	$3,823.31 ± $8,415.43	$4,777.91 ± $10,820.92	$20,809.75 ± $17,146.07	$2,667.26 ± $4,513.55	155.26 ± 1,608.25
**Aggregated costs for other services**	$6,454.81 ± $6,409.55	$6,497.90 ± $8,635.84	$5,569.58 ± $7,711.13	$6,345.16 ± $8,608.56	$3,067.41 ± $4,996.25	3,161.28 ± 6,112.04

Abbreviations ED: emergency department; GP: general practitioner; NDFP: New Drug Funding Program

Exhibit 8. List of eligible therapies for first-, second-, and third-line therapy for follicular lymphoma and marginal zone lymphoma.

Therapy Category	Regimen group	Cancer Care Ontario Regimen	First line therapy	Second line therapy	Third line therapy
**Bendamustine**	Bendamustine	*BEND	0	1	1
**BR**	BR	*BEND+RITU	1	1	1
**Bendamustine**	Bendamustine	*BENDAMUSTINE	0	1	1
**Etoposide-based**	CEOP	*CEOP	0	1	1
**Etoposide-based**	CEOP-R	*CEOP+RITU	0	1	1
**Etoposide-based**	CEP	*CEP	0	1	1
**Etoposide-based**	CEP-R	*CEP+RITU	0	1	1
**Etoposide-based**	CEP-R	*CEP-RITUXIMAB	0	1	1
**Chlorambucil-based**	Chlorambucil	*CHLO	0	1	1
**Chlorambucil-based**	CHLO+OBIN	*CHLO+OBIN	0	1	1
**Chlorambucil-based**	CHLO-RITU	*CHLO+RITU	0	1	1
**Chlorambucil-based**	CHLO-PRED	*CHLOPRED	0	1	1
**Chlorambucil-based**	Chlorambucil	*CHLORAMBUCIL	0	1	1
**Chlorambucil-based**	CHLO-PRED	*CHLORAMBUCIL-PRED	0	1	1
**Chlorambucil-based**	CHLO-RITU	*CHLORAMBUCIL-RITU	0	1	1
**Chlorambucil-based**	CHLO-RITU	*CHLORAMBUCIL-RITUX	0	1	1
**Etoposide-based**	CHOEP	*CHOEP	0	1	1
**Etoposide-based**	CHOEP-R	*CHOEP-RITUXAN	0	1	1
**CHOP**	CHOP	*CHOP	1	1	1
**R-CHOP**	R-CHOP	*CHOP+R	1	1	1

**R-CHOP**	R-CHOP	*CHOP-RITUXIMAB	1	1	1
**Gemcitabine-based**	CISPGEMC	*CISPGEMC	0	1	1
**Gemcitabine-based**	CISPGEMC	*CISPGEMC(W)	0	1	1
**Gemcitabine-based**	GEM-CARBO	*CRBPGEMC	0	1	1
**CVP**	CVP	*CVP	1	1	1
**CVP**	CVP	*CVP(PO)	1	1	1
**R-CVP**	R-CVP	*CVP+R	1	1	1
**CVP**	CVP	*CVP-IV	1	1	1
**CVP**	CVP	*CVP-PO	1	1	1
**R-CVP**	R-CVP	*CVP-RITUXIMAB	1	1	1
**Other**	Cyclophosphamide	*CYCL	0	0	1
**Other**	Cyclophosphamide	*CYCL(PO)	0	0	1
**Other**	Cyclophosphamide-prednison	*CYCL(PO)PRED	0	0	1
**Other**	Cyclophosphamide-R	*CYCL+RITU	0	0	1
**Other**	Cyclophosphamide	*CYCLO IV	0	0	1
**Other**	Cyclophosphamide	*CYCLO-PO	0	0	1
**Other**	Cyclophosphamide-prednisone	*CYCLPRED	0	0	1
**Other**	Lenalidomide-dexamethasone	*DEXALENA	0	0	1
**Other**	DHAP	*DHAP	0	1	1
**Etoposide-based**	ESHAP	*ESHAP	0	1	1
**Etoposide-based**	Etoposide	*ETOP	0	0	1
**Etoposide-based**	Etoposide	*ETOP(PO)	0	0	1
**Etoposide-based**	Etoposide-based	*ETOP-PO	0	0	1
**Etoposide-based**	Etoposide-based	*ETOPMELP(HD)	0	1	1
**Fludarabine-based**	Fludarabine-based	*FC	0	1	1

**Fludarabine-based**	Fludarabine-R-based	*FC(PO)+R	0	1	1
**Fludarabine-based**	Fludarabine-R-based	*FC+R	0	1	1
**Fludarabine-based**	Fludarabine-based	*FCM	0	1	1
**Fludarabine-based**	Fludarabine-R-based	*FCM+R	0	1	1
**Fludarabine-based**	Fludarabine-R-based	*FCM+RITU	0	1	1
**Fludarabine-based**	Fludarabine	*FLUD	0	1	1
**Fludarabine-based**	Fludarabine	*FLUD(PO)	0	1	1
**Fludarabine-based**	FLUD+R	*FLUD+R	0	1	1
**Fludarabine-based**	FCM+R	*FLUD-CYCL-MITOX-RITUX	0	1	1
**Fludarabine-based**	FCM	*FLUD-CYCLO-MITOXAN	0	1	1
**Fludarabine-based**	FC+R	*FLUD-CYCLO-RITUXIMAB	0	1	1
**Fludarabine-based**	Fludarabine	*FLUDARABINE	0	1	1
**Fludarabine-based**	Fludarabine	*FLUDARABINE PO	0	1	1
**Fludarabine-based**	FC	*FLUDARABINE-CYCLO	0	1	1
**Fludarabine-based**	FLUD+MITOX	*FLUDARABINE-MITOX	0	1	1
**Fludarabine-based**	FLUD+R	*FLUDARABINE-RITUXIMAB	0	1	1
**Gemcitabine-based**	GDP	*GDP	0	1	1
**Gemcitabine-based**	GDP-R	*GDP+RITU	0	1	1
**Gemcitabine-based**	GDP-R	*GDP-RITUXIMAB	0	1	1

**Gemcitabine-based**	GEM-CARBO	*GEM-CARBO	0	1	1
**Gemcitabine-based**	CISPGEMC	*GEM-CISP	0	1	1
**Gemcitabine-based**	CISPGEMC	*GEM-CISP-MOD	0	1	1
**Gemcitabine-based**	GEMC	*GEMC	0	1	1
**Gemcitabine-based**	GEMC	*GEMCIT	0	1	1
**Other**	Ibrutinib	*IBRU	0	0	1
**Etoposide-based**	Etoposide-based	*ICE	0	1	1
**Other**	Idelalisib	*IDEL	0	0	1
**Other**	Lenalidomide	*LENA	0	0	1
**Other**	Lenalidomide	*LENA(MNT)	0	0	1
**Other**	Lenalidomide-dexamethasone	*LENALIDOMIDE-DEXA	0	0	1
**Rituximab**	Rituximab	*RITU	1	1	1
**Rituximab**	Rituximab-M	*RITU(MNT)	1	1	1
**Rituximab**	Rituximab-M	*RITU(MNT-SC)	1	1	1
**Rituximab**	Rituximab	*RITUXIMAB	1	1	1
**Rituximab**	Rituximab	*RITUXIMAB (IT)	1	1	1
**Rituximab**	Rituximab-M	*RITUXIMAB MAINTENAN	1	1	1
**Rituximab**	Rituximab-M	*RITUXIMAB MAINTENANCE	1	1	1
**Rituximab**	Rituximab-M	*RITUXIMABMAINTEN	1	1	1
**BR**	BR	BEND+R	1	1	1
**BR**	BR	BEND+RITU	1	1	1
**CHOP**	CHOP	CHOP	1	1	1
**R-CHOP**	R-CHOP	CHOP+R	1	1	1
**R-CHOP**	R-CHOP	CHOP-RITUX	1	1	1
**R-CHOP**	R-CHOP	CHOP-RITUXIMAB	1	1	1
**CVP**	CVP	CVP	1	1	1
**R-CVP**	R-CVP	CVP+R	1	1	1
**CVP**	CVP	CVP-IV	1	1	1
**R-CVP**	R-CVP	CVP-RITUXI	1	1	1

**R-CVP**	R-CVP	CVP-RITUXIMAB	1	1	1
**Etoposide-based**	ESHAP	ESHAP	0	1	1
**Fludarabine-based**	FC+R	FC+R	0	1	1
**Fludarabine-based**	Fludarabine	FLUDARABINE	0	1	1
**Fludarabine-based**	FC	FLUDARABINE-CYCL	0	1	1
**Gemcitabine-based**	GEMC	GEMC	0	1	1
**Rituximab**	Rituximab	RITU	1	1	1
**Rituximab**	Rituximab-M	RITU(MNT)	1	1	1
**Rituximab**	Rituximab	RITUXIMAB	1	1	1
**Rituximab**	Rituximab-M	RITUXIMAB-MAINTENANCE	1	1	1
**Rituximab**	Rituximab-M	RITUXM	1	1	1

Note: 0 = not eligible, 1 = eligible
